# Investigation and analysis of influencing factors of occupational happiness among male psychiatric nurses

**DOI:** 10.1097/MD.0000000000045146

**Published:** 2025-10-10

**Authors:** Jie Liu, Mengting Zhu, Li Tao, Jing Gu, Huanhuan Deng, Li Tang, Mei Tang, Jinlan Dai, Hong Peng

**Affiliations:** a Department of Psychosomatic Medicine, Deyang People’s Hospital, Deyang, Sichuan, China; b School of Basic Medical Sciences & School of Nursing, Chengdu University, Sichuan, China; c Department General, Dujiangyan City Qingchengshan Town Health Center Taiping Branch, Sichuan, China.

**Keywords:** influence factor, male nurse, occupational happiness, psychiatry, self-esteem, social support

## Abstract

This study investigates the current status of occupational happiness among male psychiatric nurses and explores relevant influencing factors, in order to provide reference for the development of intervention measures to improve the occupational happiness of male psychiatric nurses. From June 2022 to April 2023, a questionnaire survey was conducted on 692 male psychiatric nurses using the Medical Workers’ Occupational Happiness Scale, self-esteem scale, and Social Support Scale, and the data was analyzed. The occupational happiness score of male nurses was 71.37 ± 10.76, the self-esteem score was 29.80 ± 5.99, and the social support score was 38.61 ± 9.23; The results of univariate analysis showed that there were statistically significant differences (*P* < .05) in the occupational happiness scores of male psychiatric nurses with different levels of education, nature of employment, position, personal monthly income, weekly night shifts, weekly working hours, sleep time, and whether they were specialized nurses; Pearson correlation analysis showed a positive correlation between self-esteem scores, social support scores, and occupational happiness scores, with statistically significant differences (*P* < .05); The results of multivariate analysis showed that self-esteem level, social support level, educational background, nature of employment, position, personal monthly income, daily sleep time, and whether they are specialized nurses are the main influencing factors for the occupational happiness of male psychiatric nurses, which can explain 57.8% of the total variance. The occupational happiness of male psychiatric nurses is at a moderate to low level. Male psychiatric nurses with a bachelor’s degree or above, a current position, a higher personal monthly income, sufficient sleep, a higher level of self-esteem, and a higher level of social support have a stronger sense of professional happiness. It is suggested that nursing managers should pay attention to the occupational happiness of male psychiatric nurses and develop scientific and effective strategies to improve the occupational happiness of this group.

## 1. Introduction

In the contemporary mental health care system, male healthcare professionals have gradually established a distinctive professional identity leveraging their dual advantages of physiological capabilities and psychological traits.^[1]^ In the face of special scenarios such as frequent emotional fluctuations and sudden crisis management in psychiatric nursing work, male practitioners demonstrate physical adaptability, emotional stability, and emergency decision-making abilities, enabling them to play a critical role in ensuring patient safety and maintaining treatment order.^[2]^ However, long-standing societal and cultural stereotypes regarding occupational gender roles have confined nursing work to a “female-dominated field” leading male practitioners to face identity crises. In a study covering 41 representative psychiatric hospitals in China, male nurses account for only 20.7% (approximately 1/5 of the nursing staff).^[3]^ As pointed out in the “2024 Survey Report on the Mental Health Status of Psychiatric Nurses” by the Institute of Psychology, Chinese Academy of Sciences, the anxiety level of male psychiatric nurses is significantly higher than that of female nurses. Especially regarding the risk of severe anxiety, the detection rate among male nurses reaches 15.1%, which is 8.5 percentage points higher than that among female nurses.^[4]^

The traditional notion that “a man’s ambition lies in the wider world” conflicts with the “soft” characteristics of nursing work. Nurses are stereotyped in people’s minds as gentle, compassionate, and delicate female figures. In contrast, certain inherent traits of males are exaggerated as clumsiness, poor skills, and even a lack of compassion, making them seem only capable of undertaking physical labor.^[5]^ This creates invisible barriers for this group in terms of career development pathways and recognition of social status, making it difficult for them to fully satisfy their needs for professional belonging and self-worth realization.^[6]^

Occupational happiness, as a core dimension for measuring occupational health status, encompasses individuals’ cognitive evaluations of occupational value, emotional experiences during the work process, and the extent to which occupational development goals are achieved.^[7]^ This concept includes satisfaction with basic elements such as compensation and working environment, as well as developmental needs like occupational identity and self-efficacy, ultimately pointing to the higher psychological need to achieve life meaning through occupational activities.^[8]^ Its formation process exhibits significant dynamic characteristics, influenced by both internal factors such as an individual’s emotional management capabilities and stress coping strategies, as well as external conditions such as social support networks and organizational cultural atmospheres.^[9]^ Low occupational happiness among nurses exacerbates work pressure, disrupts sleep quality, and even induces depressive emotions, significantly increasing their risk of occupational burnout.^[10]^ Additionally, it weakens professional identity and raises the turnover rate. As nurses struggle to devote sufficient energy to high-quality humanistic care, this further leads to a decline in patient care quality.^[11]^ In the context of psychiatric nursing, high-frequency emotional labor exhaustion, complex interpersonal interaction pressures, and unique work risks collectively constitute composite variables influencing occupational happiness. Enhancing occupational happiness not only requires individuals to establish a positive occupational cognitive framework but also depends on organizations building comprehensive support systems to form a virtuous cycle of synergistic development between individuals and organizations.^[12]^

Focusing on research into the occupational happiness of male psychiatric nurses has dual significance in promoting the development of the nursing profession and optimizing human resources.^[13]^ From the perspective of medical quality, enhancing practitioners’ occupational happiness experiences can directly translate into higher-quality patient care services. From the perspective of team building, strengthening occupational identity and belonging is a key pathway to stabilizing professional teams and reducing talent turnover.^[14]^ This study systematically reviews the theoretical framework of occupational happiness, combines the unique characteristics of psychiatric nursing work, and constructs a multi-dimensional analytical framework encompassing personal traits, work environment, organizational support, and social culture. Through empirical surveys and analyses of influencing factors, the study aims to develop targeted intervention strategies: these include guidance programs to help practitioners establish professional value recognition, as well as organizational management innovations to optimize the work environment and improve support systems. These research findings will provide theoretical support and practical guidance for talent cultivation, professional development, and team building in the field of psychiatric nursing, ultimately achieving the multidimensional goals of improving patient care quality, enhancing practitioners’ professional well-being, and increasing the stability of medical teams.

## 2. Materials and methods

### 2.1. Participants

Using convenience sampling, male nurses working in psychiatric wards or specialized psychiatric hospitals at all levels of comprehensive hospitals in our province were selected as the survey subjects from June 2022 to April 2023. Inclusion criteria: possessing nursing professional qualifications; engaged in psychiatric nursing work for at least 1 year; and informed consent, voluntarily participate in this study. Exclusion criteria: not on duty for more than 6 months due to further education, vacation, etc. The questionnaire adopts anonymous numbering and does not collect personal identification information such as the names or employee numbers of the research subjects. All participants are informed that the research team will strictly keep all collected information confidential and the data will only be used for this study, and they can withdraw at any time. This study has been approved by the Ethics Committee of Deyang People’s Hospital.

### 2.2. Demographic information

The demographic section comprised 13 questions addressing age, marital status, personal monthly income, nature of employment, position, daily sleep time, number of night shifts per week, and whether one is a specialist nurse.

### 2.3. Study tool

#### 2.3.1. Occupational happiness scale for medical workers (OWS-MW)

This scale is specifically designed to measure the occupational happiness of medical workers,^[15]^ with a total of 24 items and 5 dimensions (physical and mental health, value/ability expression, social support, economic income, and work environment). The total score of each item is added up to form a total score, with a total score of 24 to 120 points. The higher the score, the higher the occupational happiness, with a total score of 24 to 48 points, >48 to 96 points, and >96 to 120 points representing low, medium, and high levels of occupational happiness, respectively. This scale is mainly used to evaluate the degree of occupational happiness experienced by medical staff in their jobs. The scale demonstrates satisfactory reliability and validity^[16]^: the Cronbach α coefficient of each dimension and the total score of the scale is 0.84 to 0.97, and the test-retest reliability is 0.94 to 0.98. The Cronbach α coefficient of this scale is 0.879.

#### 2.3.2. Self-esteem scale

Originally developed by Rosenberg^[17]^ and later introduced by Yifu et al, it is a commonly used self-esteem self-assessment scale both domestically and internationally,^[18]^ with high reliability and validity.^[19]^ It consists of 10 items, each of which uses a Likert level 4 rating. The higher the total score, the higher the self-esteem level. The Cronbach α coefficient of the scale is 0.88, and the test-retest reliability is 0.82. The Cronbach α coefficient of this scale is 0.857.

#### 2.3.3. Social support rating scale (SSRS)

This scale is specifically designed to measure an individual’s social support,^[20]^ consisting of 10 items and 3 dimensions (objective support, subjective support, and utilization of support). The total score is obtained by adding up the scores of each item. The higher the score, the higher the level of social support. The total score is 11 to 22 points, 23 to 44 points, and 45 to 62 points, respectively, representing low, medium, and high levels of social support. This scale is widely used in clinical practice, and has good reliability and validity.^[21]^ The Cronbach α coefficient of the scale and its various dimensions ranges from 0.89 to 0.94, and the test-retest reliability is 0.92. The Cronbach α efficient of this scale is 0.827.

### 2.4. Methods for data collection

Prior to the formal investigation, the consent of the selected hospital’s science and education department and nursing department directors was obtained. The questionnaire was sent by the research team to the nursing department director, who then sent it to the head nurse. The head nurse then sent it to the department nurses who met the inclusion criteria. The nurses filled out the questionnaire with informed consent. All questions are mandatory and have a time limit of 20 minutes. Before analysis, all collected questionnaires were reviewed for completeness. In cases of minor missing data (fewer than 5% of total responses), the mean substitution method was applied for continuous variables and mode substitution for categorical variables. Questionnaires with more than 10% missing responses (n = 34) were excluded from final analysis to ensure data quality and reliability. Data of 692 patients was proceeded to statistical analysis.

### 2.5. Statistical analysis

Use Epidata3.0 (The Epidata Association, Odense, Denmark) to input data and perform statistical analysis using SPSS 22.0 (IBM Corporation, Armonk). The statistical description of counting data is based on frequency and composition ratio, while the statistical description of quantitative data is based on mean ± standard deviation. For binary classification variables, independent sample *t*-test is used. For multi-classification variables, analysis of variance is employed. For correlation analysis, Pearson correlation analysis test is adopted. For influencing factor analysis, linear regression analysis is utilized. A difference of *P* < .05 is considered statistically significant.

## 3. Results

### 3.1. Characteristics of the study sample

This study included 692 male psychiatric nurses, including 309 aged ≤ 30, 350 aged 31 to 39, and 33 aged ≥ 40; 251 unmarried cases and 441 married cases; 145 cases of vocational education, 547 cases of undergraduate or above; 149 individuals with a monthly income of ≤ 4000 yuan, 248 individuals with a monthly income of 4001 to 8000 yuan, and 295 individuals with a monthly income of ≥ 8001 yuan (Table 1).

**Table 1:** Sociodemographic characteristics of participants (n = 692).

Study data	N (%)
Age (yr)	
<30	309 (44.7%)
30–40	350 (50.6%)
>40	33 (4.8%)
Educational background	
College degree	145 (21.0%)
Undergraduate or above	547 (79.0%)
Employment nature	
Not in service	479 (69.2%)
In preparation	213 (30.8%)
Hospital level	
Hospitals of Grade III, Grade B and below	116 (16.8%)
Third-class hospital	576 (83.2%)
Duties	
No	551 (79.6%)
Yes	141 (20.4%)
Marital status	
Unmarried	251 (36.3%)
Married	441 (63.7%)
Titles	
Primary	505 (73.0%)
Intermediate	154 (22.3%)
Senior	33 (4.8%)
Work experience (yr)	
≤5	285 (41.2%)
6–10	186 (26.9%)
11–19	146 (21.1%)
≥20	75 (10.8%)
Personal monthly income (yuan)	
≤4000	149 (21.5%)
4001–8000	248 (35.8%)
≥8001	295 (42.6%)
Number of night shifts per week (times)	
0–1	317 (45.8%)
2	235 (34.0%)
≥3	140 (20.2%)
Weekly working hours (h)	
<40	146 (21.1%)
40–60	496 (71.7%)
>60	50 (7.2%)
Daily sleep time (h)	
<6	120 (17.3%)
6–8	521 (75.3%)
>8	51 (7.4%)
Specialist nurse	
Yes	138 (19.9%)
No	554 (80.1%)

### 3.2. Professional happiness score of male psychiatric nurses

The total scale score was 71.37 ± 10.76, with an overall average item score of 2.97 ± 0.45. Analysis of the individual dimensions revealed that the “work environment” dimension received the highest average item score (3.05 ± 0.51), closely followed by the “physical and mental health” dimension (3.03 ± 0.46). In contrast, the dimensions of “economic income” and “social support” had the lowest average item scores, both at 2.89 ± 0.49 and 2.89 ± 0.54, respectively. The score for the “value/capability manifestation” dimension (2.97 ± 0.43) was consistent with the overall average (Table 2).

### 3.3. Single factor analysis of general information and occupational happiness of male psychiatric nurses

A univariate analysis was conducted on the general data of 692 male psychiatric nurses, including age, marital status, personal monthly income, nature of employment, position, daily sleep time, number of night shifts per week, and whether they were specialized nurses. The results showed that there were statistically significant differences (*P* < .05) in the occupational happiness scores of male nurses with different educational backgrounds, nature of employment, position, monthly personal income, weekly working hours, number of night shifts per week, daily sleep time, and whether they were specialized nurses (Table 3).

**Table 2:** OWS-MW score of male psychiatric nurses (n = 692).

Dimension	Score	Average score of items
Physical and mental health	18.17 ± 2.74	3.03 ± 0.46
Value/Capability manifestation	17.84 ± 2.58	2.97 ± 0.43
Social support	14.49 ± 2.69	2.89 ± 0.54
Economic income	8.66 ± 1.46	2.89 ± 0.49
Work environment	12.21 ± 2.04	3.05 ± 0.51
Total score	71.37 ± 10.76	2.97 ± 0.45

OWS-MW = occupational well-being scale for medical workers.

**Table 3:** Single factor analysis of general information and OWS-MW of male psychiatric nurses (n = 692).

Item	Number	OWS-MW score	*t*/*F*	*P*
Age (yr)				
<30	309	71.17 ± 9.95	0.146	>.05
30–40	350	71.47 ± 11.27		
>40	33	72.09 ± 12.64		
Educational background				
College degree	145	69.77 ± 13.78	2.010	<.05
Undergraduate or above	547	71.79 ± 9.78		
Employment nature				
Not in service	479	70.60 ± 9.23	2.817	<.01
In preparation	213	73.08 ± 13.46		
Hospital level				
Hospitals of Grade III, Grade B and below	116	70.59 ± 8.34	0.398	>.05
Third-class hospital	576	71.52 ± 11.18		
Duties				
No	551	70.86 ± 9.50	2.434	<.05
Yes	141	73.33 ± 14.55		
Marital status				
Unmarried	251	71.24 ± 9.88	0.219	>.05
Married	441	71.43 ± 11.24		
Titles				
Primary	505	71.27 ± 10.43	0.112	>.05
Intermediate	154	71.53 ± 11.43		
Senior	33	72.09 ± 12.65		
Work experience (yr)				
≤5	285	71.28 ± 9.89	0.342	>.05
6–10	186	71.17 ± 10.94		
11–19	146	71.17 ± 11.64		
≥20	75	72.55 ± 11.80		
Personal monthly income (yuan)				
≤4000	149	68.24 ± 12.08	17.962	<.01
4001–8000	248	70.07 ± 9.07		
≥8001	295	74.03 ± 10.78		
Number of night shifts per week (times)				
0–1	317	73.65 ± 10.75	23.699	<.01
2	235	71.26 ± 11.10		
≥3	140	66.37 ± 8.26		
Weekly working hours (h)				
<40	146	74.62 ± 11.57	8.833	<.01
40–60	496	70.59 ± 10.42		
>60	50	69.60 ± 9.94		
Daily sleep time (h)				
<6	120	67.95 ± 12.74	9.040	<.01
6–8	521	71.84 ± 9.42		
>8	51	74.59 ± 15.79		
Specialist nurse				
Yes	138	79.04 ± 12.59	10.010	<.01
No	554	69.45 ± 9.33		

OWS-MW = occupational well-being scale for medical workers.

### 3.4. The relationship between self-esteem, social support, and occupational happiness among male psychiatric nurses

The findings reveal strong positive correlations between occupational happiness in male psychiatric nurses and both self-esteem (*R* = 0.643) and perceived social support (*R* = 0.621). These high correlation coefficients (both statistically significant at *P* < .01) indicate that higher levels of self-esteem and stronger social support networks are robustly associated with greater job satisfaction in this group. Practically, this suggests that enhancing nurses’ personal sense of competence and fostering supportive work/environmental relationships could be highly effective strategies for improving their workplace well-being and potentially reducing burnout in this demanding specialty.

### 3.5. Multivariate analysis of factors influencing the occupational happiness of male psychiatric nurses

Conduct unconditional multiple linear regression analysis using statistically significant variables in univariate analysis and occupational happiness of male psychiatric nurses as independent and dependent variables. The results showed that education level, nature of employment, position, personal monthly income, daily sleep time, whether a specialist nurse, self-esteem, and social support were factors affecting the occupational happiness of male nurses. This result is similar to Wang Chao’s research findings^[22]^ (Table 4).

**Table 4:** Multivariate analysis of factors influencing the OWS-MW of male psychiatric nurses (n = 692).

Item	*B*	SE	β	*t*	*P*
Educational background	−1.976	1.000	−0.075	−1.976	<.05
Employment nature	−3.223	0.778	−0.138	−4.144	<.01
Duties	6.927	1.098	0.260	6.310	<.01
Personal monthly income (yuan)	2.942	0.453	0.211	6.494	<.01
Daily sleep time (h)	1.356	0.397	0.061	2.264	<.05
Specialist nurse	−2.727	0.581	−0.101	−3.135	<.01
SES	0.742	0.070	0.413	10.666	<.01
SSRS	0.415	0.050	0.356	8.293	<.01
Constant	29.052	3.756	–	7.735	<.01

*R*^2^ = 0.584, adjusted *R*^2^ = 0.578, *F* = 95.737, *P* < .01.

OWS-MW = occupational well-being scale for medical workers, SES = self-esteem scale, SSRS = social support rating scale.

## 4. Discussion

### 4.1. Current status of occupational happiness among male psychiatric nurses

The results of this survey show that the occupational happiness score of male psychiatric nurses is 71.37 ± 10.76, with an average score of 2.97 ± 0.45, which is at a moderate to low level.^[23]^ This research result is lower than those of Chen Lingli et al^[24]^ and Liu Xiaohong et al,^[25]^ possibly because they did not consider the gender factor and the impact of social prejudice suffered by male nurses on their own occupational happiness. The reason for this is that psychiatric diseases and the special nature of psychiatric wards can easily lead to a lower perceived social status among male nurses. In addition, the public’s sense of identity with male nurses and psychiatric nursing work is lower, resulting in higher work pressure and lower levels of occupational happiness among male psychiatric nurses.^[26,27]^ Therefore, it is necessary to strengthen the professional identity of the public towards male nurses and the professional identity of male psychiatric nurses towards psychiatric nursing work, which will have a good promoting effect on the professional happiness of male psychiatric nurses. These findings can be further interpreted through Herzberg two-factor theory, which distinguishes between “hygiene factors and “motivational factors,”^[28]^ providing a theoretical framework for analyzing the influencing factors below.

### 4.2. Analysis of influencing factors on occupational happiness of male psychiatric nurses

The results of this survey show that with the increase of education, the level of occupational happiness of male psychiatric nurses is also improving. The reason behind this is that individuals with higher education levels are more able to utilize their higher knowledge and skills to promote their own growth.^[29]^ In addition, individuals with higher education levels are often more likely to receive the attention and training of their units and leaders, and often have more opportunities for learning and promotion.^[30]^ Therefore, clinical managers should strengthen the training of male psychiatric nurses with lower education levels, expand continuing education channels according to relevant policies, encourage educational advancement, and ultimately enhance their self-affirmation of the nursing profession, ultimately enhancing their sense of career happiness.

The results of this survey show that the occupational happiness level of male psychiatric nurses increases with the increase of monthly income. The reason behind this is that salary levels not only provide individuals with material security, but also directly reflect the work effort and return, which can affect the perception of income satisfaction among male psychiatric nurses,^[31]^ thereby directly affecting their sense of career happiness.^[12]^ Therefore, it is recommended that hospital managers establish a reward mechanism to appropriately increase the salary level of male psychiatric nurses, guide their work values to shift towards a positive and healthy direction, and thereby enhance their professional identity.

The results of this survey show that male nurses in the psychiatric department who are currently employed have higher scores of occupational happiness than those who are not employed, and the difference is statistically significant (*P* < .05). The reason behind this is that under the same working environment and conditions, male psychiatric nurses who are employed often enjoy higher salary levels, welfare benefits, social security, and more opportunities for further education, development, promotion, and competition compared to non-employed nurses. Therefore, they have a stronger sense of career happiness.^[32]^ This also suggests that hospital managers should continuously improve their work allocation system, increase the level of benefits and benefits for non-hired nurses, narrow the gap, implement equal pay for equal work, and strive to achieve a balance between the efforts and rewards of nurses.^[33]^

The results of this survey showed that male psychiatric nurses with positions had higher occupational happiness scores than those without positions, and the difference was statistically significant (*P* < .05). The reason for this is that the position to some extent reflects an individual’s overall ability level. The higher the position, the richer the work experience, the higher the salary and benefits, and the easier it is to meet material needs.^[34]^ At the same time, as leaders of the nursing team, those in positions can participate more in decision-making, have a stable social status and a wide social network, and have a strong sense of career achievement, Therefore, their level of occupational happiness is also relatively high.^[35]^ This suggests that clinical managers should provide more encouragement and care to male psychiatric nurses without positions in their work, and provide them with more opportunities to participate in departmental and hospital affairs, improve their sense of career achievement, and thus enhance their level of career happiness.

The results of this survey show that the shorter the daily sleep time, the lower the level of professional happiness of male psychiatric nurses. Sleep quality is one of the important factors affecting individual physical and mental health,^[36]^ and sufficient sleep is the source of energy and physical and mental pleasure for nurses. Poor sleep quality can lead to negative emotions of boredom and dissatisfaction among nurses, reduce work efficiency, and even lead to the occurrence of errors and accidents.^[37]^ Therefore, it is recommended that nursing managers adopt a humanized management model, flexible scheduling, and reasonable arrangement of learning and meeting time to ensure the sleep time of nurses as much as possible.

The results of this survey show that male psychiatric nurses who are specialized nurses have higher occupational happiness scores than those who are not specialized nurses, and the difference is statistically significant (*P* < .05). Upon investigation, obtaining certification as a specialist nurse is an important factor affecting the self-career planning of male psychiatric nurses, and the self-career planning of specialist nurses is relatively good.^[38]^ Through the training of specialized male psychiatric nurses, their specialized nursing abilities in the field of psychiatry have been further improved. They are more likely to excel in solving difficult nursing problems in the field, and their professional value has been better reflected.^[39]^ Therefore, specialized nurses have a stronger sense of professional happiness. Department managers should provide more channels for specialized nurse training or learning to male psychiatric nurses, encourage them to participate more in psychiatric specialty training lectures and obtain specialty certificates, in order to enhance their professional happiness.

The results of this survey show that the self-esteem level of male psychiatric nurses is positively correlated with their occupational happiness score (*R* = 0.643, *P* < .01). The reason behind this is that male psychiatric nurses with high self-esteem levels are able to actively cope with stress, have positive interpersonal relationships, and experience more self-worth. This positive emotion buffers the adverse effects of work pressure, resulting in higher occupational happiness.^[40]^ This suggests that managers should attach importance to the psychological construction of male psychiatric nurses, take appropriate psychological intervention measures to improve their self-esteem. For example, sharing sessions can be organized weekly or monthly basis to encourage male nurses to express their thoughts and concerns, thus enhancing their professional happiness and promoting their better personal value.^[41]^

The results of this survey show that the social support level of male psychiatric nurses is positively correlated with their occupational happiness score (*R* = 0.621, *P* < .01), indicating that the more social support male psychiatric nurses have, the higher their occupational happiness. The reason behind this is that good social support can not only provide material assistance to individuals, but also provide psychological support, thereby helping them alleviate work pressure and improve their sense of career happiness.^[42]^ This suggests that nursing managers should understand and support male psychiatric nurses as much as possible, providing them with more social support from colleagues. Male psychiatric nurses themselves should actively integrate into society, strengthen communication with family, friends, and colleagues, and improve the utilization of existing social support, establishing positive and effective social coping methods.

Hygiene factors, which prevent dissatisfaction, include personal monthly income, nature of employment, and daily sleep time.^[28]^ Their inadequacy, such as low income, unstable contracts, or insufficient sleep, directly reduces happiness, but their sufficiency serves as a basic prerequisite rather than a direct driver of positive satisfaction. In contrast, motivational factors, which enhance intrinsic satisfaction, include educational background, job position, specialist nurse status, and self-esteem.^[28]^ These factors satisfy needs for professional growth, achievement, and recognition, thereby directly boosting the sense of occupational happiness. This theoretical distinction clarifies that improving the occupational happiness of male psychiatric nurses requires both optimizing hygiene factors to eliminate dissatisfaction and strengthening motivational factors to foster intrinsic satisfaction, providing a targeted direction for intervention strategies.

## 5. Limitations

This study has several limitations that should be acknowledged. First, this study is a cross-sectional study. Due to its design characteristics, it is impossible to directly infer the causal relationship between variables from the research results. Second, the samples of this study are all sourced from the same province, which may lead to limitations in regional representativeness and, to some extent, restrict the generalizability of the research results. Finally, all variables in this study were collected through self-reporting methods, which may introduce response bias and affect the accuracy and objectivity of the data to a certain degree. Based on this, future research could further conduct a national multi-center longitudinal study, incorporate more representative and diverse samples, and track the long-term effects of intervention measures on the occupational happiness of male psychiatric nurses to make up for the deficiencies of the current study.

## 6. Implications

Existing research has limited attention to the occupational happiness of psychiatric nurses. Research specifically focusing on male psychiatric nurses is even rarer. Male nurses are a sub-group that has not been fully explored in nursing research for a long time. In the past decade, the number of domestic papers related to male nurses published each year is <100, and the research focus is mostly on male nurses in operating rooms and emergency departments.^[43]^ Research on male psychiatric nurses is particularly rare.

This study takes male psychiatric nurses as a unique entry point to break through the boundaries of existing research. Different from previous studies that generalize nursing staff without detailed gender-difference analysis, this study reveals the gender-specific challenges faced by male psychiatric nurses. The research not only explores the influencing factors of job happiness but also deeply analyzes the internal mechanisms by which these factors act on happiness, providing a reference for nursing managers to develop intervention measures. This is not only helpful for enhancing male nurses’ professional identity and reducing their turnover rate, but also can attract men to choose the psychiatric nursing profession., ultimately expanding the team of psychiatric nursing staff.

## 7. Conclusion

In summary, the results of this study indicate that the occupational happiness of male psychiatric nurses is at a moderate to low level and needs further improvement. A higher personal monthly income, sufficient sleep, a higher level of self-esteem, and a higher level of social support have a stronger sense of professional happiness. Male nurses are an indispensable main force in psychiatric nursing work, and their level of occupational happiness is related to the overall effectiveness of nursing work. Therefore, paying attention to the level of occupational happiness of male psychiatric nurses and adopting targeted improvement strategies can help improve their occupational happiness. This is crucial for fully leveraging the advantages of male psychiatric nurses and promoting the stability of the psychiatric nursing team.

## Acknowledgments

The authors would like to thank the nursing department directors, head nurses, and male psychiatric nurses from various hospitals who participated in the survey.

## Author contributions

**Conceptualization:** Jie Liu, Li Tao.

**Data curation:** Jie Liu, Jing Gu, Jinlan Dai.

**Formal analysis:** Jie Liu, Huanhuan Deng, Hong Peng.

**Investigation:** Huanhuan Deng, Li Tang, Mei Tang, Jinlan Dai.

**Methodology:** Jie Liu, Li Tao, Jing Gu, Huanhuan Deng, Li Tang, Mei Tang.

**Project administration:** Jie Liu, Jing Gu.

**Software:** Mengting Zhu.

**Supervision:** Jie Liu, Li Tao, Hong Peng.

**Validation:** Jie Liu, Mengting Zhu, Li Tao.

**Visualization:** Jie Liu, Huanhuan Deng.

**Writing – original draft:** Jie Liu, Li Tao, Jinlan Dai.

**Writing – review & editing:** Jie Liu, Mengting Zhu, Jinlan Dai.
